# Isolation and Characterization of Phenolic Compounds from the Leaves of *Salix matsudana*

**DOI:** 10.3390/molecules13081530

**Published:** 2008-08-03

**Authors:** Xiang Li, Zhi Liu, Xin-feng Zhang, Li-juan Wang, Yi-nan Zheng, Chang-chun Yuan, Guang-zhi Sun

**Affiliations:** 1 College of Chinese Medicinal Materials, Jilin Agricultural University, Changchun, 130118, P.R. China; E-mails: lixiang@bjmu.edu.cn (Xiang Li); ljwang@yahoo.com (Li-juan Wang); zhenyinan@tom.com (Yi-nan Zheng); 2 Institute of Agricultural Modernization, Jilin Agricultural University, Changchun, 130118, P.R. China; E-mails: jlndxdhs@126.com; ccyuan@yahoo.com; 3 College of Forestry and Biotechnology, Zhejiang Forestry University, Hangzhou, 311300, P.R. China; E-mail: xhzhang@hotmail.com

**Keywords:** *Salix matsudana*, matsudone A, flavonoid, cyclooxygenase inhibition.

## Abstract

A bioassay-guided *in vitro* screen has revealed that a 70% methanol extract of the leaves of *Salix matsudana* shows considerable inhibitory activity against cyclo-oxygenases (COX-1 and COX-2). A subsequent phytochemical study led to the isolation of a new flavonoid, matsudone A (**1**), together with five known flavonoids – luteolin (**2**), isoquercitrin (**3**), 7-methoxyflavone (**4**), luteolin 7-*O*-glucoside (**5**), 4',7-dihydroxyflavone (**6**) – and two phenolic glycosides, leonuriside A (**7**) and piceoside (**8**). Their structures were elucidated on the basis of extensive 1D- and 2D-NMR studies, high resolution ESI mass spectroscopic analyses and comparisons with literature data. The isolated compounds **1**-**8** were tested for their inhibitory activities against COX-1 and COX-2. Compounds **1**, **5** and **6** were found to have potent inhibitory effect on COX-2 and compounds **3**-**5** exhibited moderate inhibition against COX-1.

## Introduction

**Figure 1:**
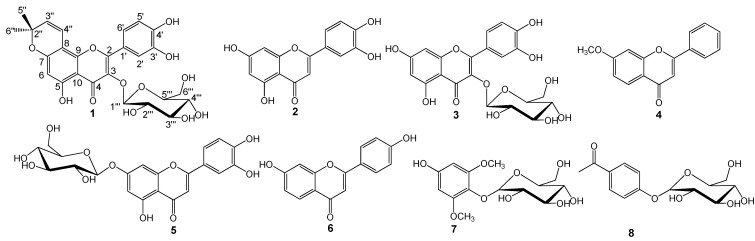
Flavonoids and phenolic glycosides isolated from the leaves of *Salix matsudana*.

*Salix matsudana* Koidz is a small to medium-sized upright spreading tree reaching up to about 30 feet in height and a 15-foot-spread, distributed around the world [1]. In the Traditional Chinese Medical (TCM) literature it was identified as a plant with alexipharmic and antiphlogistic properties, with several pharmacological applications, such as in the treatment of jaundice, hepatitis, rheumatism, and arthritis, as well as eczema [2,3]. Previous phytochemical examinations showed that it produced several kinds of flavonoids, phenolic compounds and diterpene-γ-lactones [4,5,6]. As part of our investigations on the chemical constituents and pharmacological activities of *Salix* species [7,8,9,10], a new flavonoid, named matsudone A (**1**), has been isolated from a 70% methanol extract of the leaves of *Salix matsudana*, along with seven known compounds **2**-**8** (Figure 1). Their structures were elucidated on the basis of high resolution (HR) ESI-MS, ^1^H- and ^13^C-NMR, together with 2D-NMR spectroscopic analyses. The isolated compounds were tested in a cyclooxygenase (COX-1 and COX-2) bioassay and compounds **1**, **5** and **6** were found to have potent inhibitory effect on COX-2, while compounds **3**-**5** exhibited moderate inhibition against COX-1. 

## Results and Discussion

### Characterization of compounds **1–8**

Compound **1** was isolated as a pale yellow amorphous powder, and its molecular formula was determined as C_26_H_26_O_12_ on the basis of HR-ESI-MS (*m/z* 531.1517 [M+H]^+^, *calcd.* 531.1503) and NMR data (Table 1), implying fourteen degrees of unsaturation. The UV (MeOH) bands at 246, 285 (sh), 325 nm suggested a flavonol skeleton. The ^13^C-NMR and DEPT spectra displayed two methyls, one methylene, eleven methines, and twelve quaternary carbons. The phenolic region NMR data of **1** was similar to those of the known compound, 7, 8-(2'',2''-dimethylpyrano)-5,3',4'-trihydroxy-3-methoxyflavone (Table 1), which had been previously characterized and isolated from the medicinal plant *Hypericum Japonicum* [11]. The only difference between the two compounds concerned the substitution at C-3, since while the known compound had a methoxyl group at this position, compound **1** was substituted by a sugar group. 

**Table 1:** The NMR data of compound **1** ^a^.

Position	^1^H-NMR	*J* (Hz)	^13^C-NMR	DEPT	COSY	HMBC	^13^C-NMR ^b^
**2**	-	-	155.2	C	-	-	155.4
**3**	-	-	142.7	C	-	-	137.2
**4**	-	-	178.9	C	-	-	177.5
**5**	-	-	155.0	C	-	-	154.8
**6**	6.40, *s*	-	95.3	CH	-	C-8, C-10	94.1
**7**	-	-	160.0	C	-	-	158.0
**8**	-	-	105.1	C	-	-	103.8
**9**	-	-	153.9	C	-	-	154.7
**10**	-	-	105.6	C	-	-	104.6
**1’**	-	-	122.1	C	-	-	120.0
**2’**	7.67, *d*	1.7	117.2	CH	H-6'	C-2, C-3', C-4', C-6'	115.1
**3** **’**	-	-	145.3	C	-	-	144.6
**4** **’**	-	-	147.6	C	-	-	148.3
**5** **’**	6.95, *d*	8.5	114.7	CH	H-6'	C-1', C-3'	115.0
**6** **’**	7.38, *dd*	8.5, 1.7	120.7	CH	H-2', H-6'	C-1', C-2, C-2', C-4'	120.0
**2”**	-	-	78.1	C	-	-	77.4
**3”**	5.57, *d*	10.0	125.7	CH	H-4''	C-5''', C-6''', C-7, C-8	128.5
**4”**	6.56, *d*	10.0	116.3	CH	H-3''	C-5''', C-6''', C-7, C-9	113.9
**5”**	1.47, *s*	-	27.3	CH_3_	-	C-2''', C-3'''	27.2
**6”**	1.47, *s*	-	27.3	CH_3_	-	C-2''', C-3'''	27.2
**1’’’**	5.15, *d*	7.6	102.2	CH	H-2'''	C-3, C-3'''	-
**2’’’**	3.18, *m*	-	72.7	CH	-	-	-
**3’’’**	3.2 – 3.5, *m*	-	77.5	CH	-	-	-
**4’’’**	3.2 – 3.5, *m*	-	70.6	CH	-	-	-
**5’’’**	3.2 – 3.5, *m*	-	76.9	CH	-	-	-
**6’’’**	3.65, *d*	11.5	62.1	CH_2_	H-6'''	C-4''', C-5'''	-
**-OMe**	-	-	-	-	-	-	59.1

^a^ Compound **1** was measured in DMSO-*d*_6_ and chemical shifts are expressed in ppm; ^b^ ^13^C-NMR data (in DMSO-*d*_6_) of 7,8-(2''', 2'''-dimethylpyrano)-5, 3', 4'-trihydroxy-3-methoxyflavone [11].

**Figure 2:**
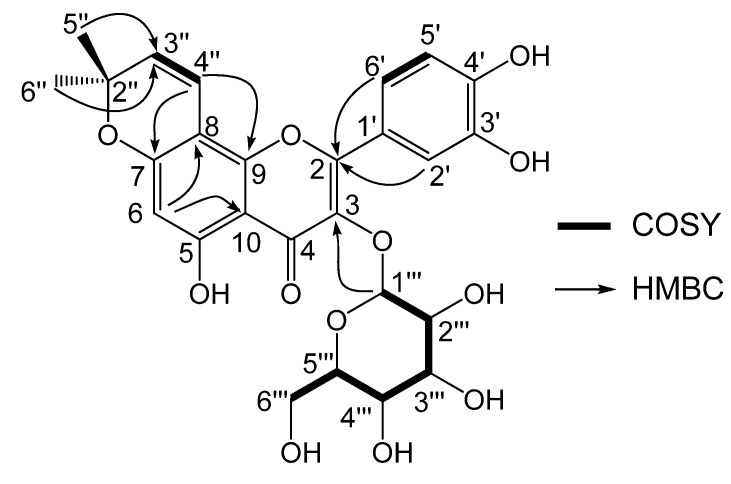
Partial HMBC and COSY correlations of compound 1.

This finding was supported by ^13^C-NMR data which showed a group of sugar signals at *δ*_C_ 102.2 (d, C-1'''), *δ*_C_ 77.5 (d, C-3'''), *δ*_C_ 76.9 (d, C-5'''), *δ*_C_ 72.7 (d, C-2'''), *δ*_C_ 70.6 (d, C-4''') and *δ*_C_ 62.1 (t, C-6'''). This sugar moiety was identified as glucose on the basis of an acid hydrolysis reaction, whose reaction product showed same *R_f_* on the TLC plate as an authentic glucose reference. The coupling constant *J*_H1''', H2'''_ (7.6 Hz) of **1** indicated a *β*-glucose. The absolute configuration of the *β*-glucose was further determined to be *β*-d-glucose by chiral GC analysis. Meanwhile, H-1''' (*δ* 5.15) showed HMBC correlation with *δ*_C-3_142.7 (Figure 2), confirming that the glucose moiety was connected to the C-3 position. Thus, compound **1** was determined to be 7,8-(2'', 2''-dimethylpyrano)-5, 3', 4'-trihydroxy-flavone-3-*O*-*β*-d-glucoside, to which we have given the name matsudone A. 

The known compounds **2-8** were identified on the basis of comparison of their NMR data with that found in the literature [12,13,14,15,16,17,18]. The sugar moieties of compounds **3**, **5**, **7** and **8** were determined by the acid hydrolysis method, as mentioned in the Experimental section. The results showed that all the above compounds contained the same sugar moiety, *β*-d-glucose.

**Figure 3:**
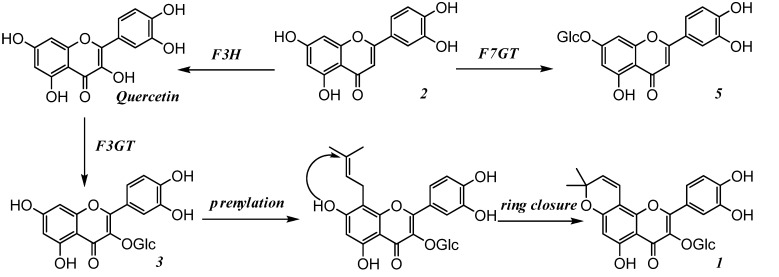
Proposed biosynthetic pathway of matsudone A (**1**) and isolated flavonoids **2**, **3** and **5**.

Flavonoids are among the best-characterized plant secondary metabolites in terms of chemistry, mechanism of coloration, biochemistry, genetics and molecular biology [19]. In the present study, we isolated several flavonoids, **1**-**6**, for which the biosynthetic pathway shown in Figure 3 may be proposed. 

Briefly, luteolin (**2**) could afford the common flavonoids quercetin and luteolin 7-*O*-glucoside (**5**), via reactions catalyzed by the enzymes flavonoid 3-hydroxylase (*F3H*) and flavonoid 7-glucosyl-transferase (*F7GT*), respectively. Quercetin could be further catalyzed by flavonoid 3-glucosyl-transferase (*F3GT*) to form isoquercetrin (**3**), and compound **1** could be formed after prenylation and ring closure. Although to our knowledge no publications have reported flavonoid pathway related genes in *Salix matsudana*, our results strongly suggested that the speculated enzymes (*F3H*, *F7GT*, *F3GT* and *prenyltransferase*) exist in this spp*.*

### Bioactivity Results

The isolated compounds were tested for their inhibitory activities against cyclooxygenase (COX-1 and COX-2, Table 2). Compounds **1**, **5** and **6** were found to have potent inhibitory effect on COX-2, with IC_50 _values (µM) of 27.3, 39.1 and 58.8, respectively; compounds **3**-**5** exhibited moderate inhibition against COX-1, with IC_50_ values (µM) of 102.7, 99.1 and 92.3, respectively. Interestingly, the new isolated matsudone A (**1**) showed considerable COX-2 inhibition activity (IC_50_ = 27.3 µM) compared to the positive control aspirin (IC_50_ = 19.0 µM), making it a good candidate for further consideration as an anti-inflammatory prodrug.

**Table 2:** IC_50_ data of isolated compounds as inhibitors of COX-1 and COX-2^a^.

#	1	2	3	4	5	6	7	8	Aspirin
**IC_50_ of COX-1 (*µM*)**	153.1	I.A.^b^	102.7	99.1	92.3	I.A.	195.4	216.9	21.7
**IC_50_ of COX-2 (*µM*)**	27.3	I.A.	I.A.	169.0	39.1	58.8	199.7	I.A.	19.0

^a^ Values are means of three determinations. ^b^ I.A.: Inactive.

## Conclusions

In summary, a novel flavonoid, matsudone A (1), has been isolated from leaves of *Salix matsudana*, together with seven known compounds, **2**-**8**. The isolated compounds **1**-**8** were tested for their inhibitory activities against COX-1 and COX-2. Compounds **1**, **5** and **6** were found to have potent inhibitory effects on COX-2 and compounds **3**-**5** exhibited moderate inhibition against COX-1.

## Experimental

### General

The ^1^H- and ^13^C-NMR spectra were measured on a Bruker Avance DRX 500 NMR spectrometer, using TMS as an internal standard. Chemical shifts (*δ*) are expressed in parts per million (ppm), with the coupling constants (*J*) reported in Hertz (Hz). The ESI-MS spectra were recorded on a triple quadrupole mass spectrometer Quattro (VG Biotech, Altrincham, England) and the HRESI-MS spectra on a Bruker FT-ICRMS spectrometer. Column chromatographies were carried out with silica gel 60 M (200-300 mesh), Lichrospher RP-18 (20 μm) and Sephadex LH-20 (Pharmacia); TLC was performed on silica gel plates (Macherey-Nagel, SilG/UV_254_, 0.20mm), with spots detected by UV_254_ and anisaldehyde/H_2_SO_4_ (10%). HPLC was performed on an Agilent 1100 instrument.

### Chemicals and reagents

COX-1 and COX-2 were purchased from Cayman Chemical (Michigan, US). The ^14^C-labeled arachidonic acid (>200 µCi, NEN) was purchased from New England Nuclear Co. (Boston, US). Other chemicals and reagents were purchased from the Chinese Chemical Group (Beijing, P.R. China). 

### Extraction and isolation

Leaves of *Salix matsudana* was collected on the campus of Jilin Agricultural University (JLAU) and identified by one of the authors, Prof. Yi-nan Zheng. A voucher specimen (HLY-06-01) has been deposited in the Laboratory of Medicinal Chemistry, JLAU. Crude powdered *Salix matsudana* leaves (6.5 kg) were extracted with 70% MeOH (20 L) at room temperature for 48 h, and the extract concentrated to give a dark brown residue (850 g). This residue was blended with silica gel and directly subjected to chromatography on a silica column, eluted with gradient mixture of CHCl_3_-MeOH-H_2_O (9:1, 5:1 and 1:1), to yield five fractions (F_A_ - F_E_). The fourth fraction, F_D_, was subjected to silica gel chromatography, eluted with CHCl_3_-MeOH-H_2_O = 7:2:0.1, then further purified by semi-preparative HPLC (gradient elution of 5% aqueous MeOH to 100% MeOH) to afford compounds **1** (7 mg) and **7** (12 mg). The fifth fraction, F_E_, was subjected to ODS RP-18 column chromatography eluted with 80% MeOH to afford compounds **3** (106 mg), **6** (12 mg) and **2** (49 mg). The third fraction, F_C_, was subjected to further chromatography on a Sephadex LH-20 column, with 70% MeOH elution, to afford compounds **4** (7 mg), **5 **(135 mg) and **8** (26 mg).

### Acid hydrolysis of compounds **1**, **3**, **5**, **7** and **8**

Compounds **1**, **3**, **5**, **7** and **8** (each 2.0 mg) were refluxed with 6 N HCl (5 mL) at 100 ºC for 2 h. Each mixture was extracted with CHCl_3_ to afford the corresponding aglycone, and the aqueous layer was neutralized with Na_2_CO_3_ and filtered. The aqueous layer was dried under vacuum and the residue was re-dissolved in H_2_O for sugar analysis by TLC with *n*-BuOH–HOAc–H_2_O (4:1:2) as the solvent. The sample spots were detected by spraying aniline hydrogen phthalate reagent (100 mL *n*-BuOH saturated by H_2_O, 0.96 g aniline and 1.66 g phthalic acid) and heating. Authentic samples of glucose, xylose and rhamnose were used as standards. The absolute configuration of the glucose detected was further determined by chiral GC analysis using a SatoChrom GC and a 0.25 mm x 25 m Hydrodexb-6-TBDM chiral capillary column (Macherey-Nagel, Germany). *β*-d-glucose was used as an authentic GC standard. The aqueous layer residues mentioned above were re-suspended in dichloromethane (1 mL), and trifluoroacetic anhydride (50 µL) was added. The mixtures were allowed to react at room temperature overnight and dried under a stream of nitrogen at room temperature. The sugar derivatives were separated using the following temperature program: inlet temperature was set at 240 °C, with hydrogen carrier gas and a 1/20 split, using nitrogen makeup gas. Column temperatures started at 120 °C, ramped to 220 °C at 50 °C min^–1^ and were maintained for 12 min.

*Matsudone A* (**1**). Obtained as a pale yellow amorphous powder; 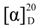 -59.1 (*c* 0.5, MeOH); mp 349.1 – 349.9 ºC; UV (MeOH) λ_max_: 246, 285 (sh), 325 nm; HRESI-MS [+]: *m/z* = 531.1517 [M+H]^+^ (*calcd*. for C_26_H_27_O_12_, 531.1503); ^1^H and ^13^C-NMR data, see Table 1.

### Effect on Cyclooxygenase-1 and -2

The effect of the tested compounds on cyclooxygenase-1 and -2 (COX-1 and -2) was determined by measuring PGE_2_ production. Generally, the reaction mixtures were prepared in Tris-HCl buffer (pH 8.0), containing hematin (1.5 µM), glutathione (300 µM), epinephrine (300 µM), enzyme (COX-1 or COX-2, 50 µL) and various concentrations of isolated compounds. 1-^14^C Arachidonic acid (10 µL) was added to start the reaction. The mixture was incubated for 30 min at 37 ºC, then, the reaction was terminated by adding the reaction mixture (20 µL) to 30 µM indomethacin (200 µL). Arachidonic acid and its radio-labeled metabolites were separated and determined by reversed-phase HPLC using a Berthold radioactivity monitor. Inhibition refers to reduction of PGE_2_ formation, in comparison to a blank run without inhibitor. The results are means of three independent experiments. Aspirin was used as a positive control.
